# The Effect of the COVID-19 Pandemic and Lockdown on Operative Traumatic Brain Injury in Northern Virginia

**DOI:** 10.7759/cureus.44746

**Published:** 2023-09-05

**Authors:** Thaddeus J Leiphart, James Leiphart

**Affiliations:** 1 Neurosurgery, Inova Health System, Falls Church, USA; 2 Biology, St. Albans School, Washington, USA; 3 Neurosurgery, University of Virginia School of Medicine, Falls Church, USA

**Keywords:** surgery, lockdown, elderly, tbi, pandemic, covid-19

## Abstract

Introduction: COVID-19 is a disease that causes flu-like symptoms and difficulty breathing. Emerging in 2019, the COVID-19 pandemic has affected the entire world through restrictions and lockdowns. Multiple studies have compared the effects of COVID-19 on different types of head trauma, with each one producing different results. The goal of this study was to use state and hospital data to determine whether the COVID-19 pandemic had a significant impact on surgeries for traumatic brain injury (TBI).

Methods: Public state data on COVID-19 incidence, sourced from the Virginia Department of Health, was compared to hospital data of 352 patients receiving surgeries for TBI from a single major level-one trauma hospital in Northern Virginia. We used data from the three years before COVID-19 and the two years during the pandemic, using t-tests and Pearson correlation to analyze the data. This is a retrospective case review study on the number and age of patients receiving TBI surgery from March 2017 through February 2022 at Inova Fairfax Hospital in Northern Virginia to determine the impact of the COVID-19 pandemic on these factors.

Results: When comparing the data, there was a 60% reduction in cases of operative TBI during the peak months of COVID-19 compared to the same months in previous years (p<0.005). Comparing data on the number of Virginia and Northern Virginia COVID-19 cases and data on the age of individuals undergoing TBI surgery four weeks later showed a statistically significant negative correlation (p<0.05) in which the average age of individuals undergoing TBI surgery was lower in the four-week block following a four-week block of increased COVID-19 incidence.

Conclusion: Our findings indicate a correlation between the period of decreased activity from COVID-19 restrictions in Virginia and a decline in both the number of TBI surgeries and the age of individuals undergoing these surgeries.

## Introduction

A coronavirus emerged in December 2019 from the city of Wuhan, China [1]. Formally named COVID-19 by WHO, it is a viral illness belonging to the order Nidovirales [1,2]. On March 11, 2020, the outbreak of COVID-19 was declared a pandemic by WHO [3]. The COVID-19 pandemic was responsible for a global lockdown and has resulted in more than 6 million deaths worldwide [4]. In Virginia, the most stringent period of lockdown restrictions started on March 13, 2020, with all K-12 schools. On March 23, 2020, it was announced that all public schools would be closed for the rest of the school year as well as recreational businesses. On March 30, 2020, a statewide stay-at-home order was issued by the governor. This period of increased restrictions lasted until July 1, 2020, when phase-three reopening began in Virginia [5]. COVID-19 and the resulting lockdown have also had multiple impacts on other disease processes and the delivery of care at medical centers.

Traumatic brain injury (TBI) is widespread, being one of the leading causes of death globally [6,7]. It is estimated that about 69 million individuals experience a TBI each year worldwide [8]. The most common sources of TBI are falls and motor vehicle collisions [9]. TBI can be classified as mild, moderate, or severe based on the patient’s presenting Glasgow Coma Scale, with many patients affected by severe TBI continuing to demonstrate cognitive and psychosocial issues [9,10].

Several studies have shown an overall decrease in the incidence of TBI during the COVID-19 pandemic compared to prior years [11-19], whereas other studies have demonstrated an overall increase in this same statistic at their respective institutions [20-25]. In addition, there have been studies that have shown neither a statistically significant increase nor decrease in the incidence of TBI during the COVID-19 pandemic compared to prior years [26,27]. The goal of this study was to analyze the effects of COVID-19 on the incidence of operative TBI at our institution in Northern Virginia. We expected to see an overall decrease in operative TBI at our institution during the COVID-19 pandemic due to lockdown restrictions indirectly reducing major sources of TBI such as motor vehicle collisions [9].

## Materials and methods

To determine the effects of the COVID-19 pandemic and its subsequent lockdown on the incidence of TBI surgeries performed in Northern Virginia, we compared hospital data from Inova Fairfax Hospital on patients receiving surgery for TBI to publicly available Virginia State data on new COVID-19 infections. We determined the number of TBI surgeries performed and the average age of the TBI surgery patients by month at our institution in the three years before the COVID-19 pandemic (March to February of 2017-2018, 2018-2019, and 2019-2020) and the two years following its start (March to February of 2020-2021 and 2021-2022). We chose to analyze the first two years following the outbreak of COVID-19 as this period of time represents the most significant impact of COVID-19 on the population, and we selected the three years prior for comparison to minimize the effects of annual variations. We compared the number of TBI surgeries performed at Inova Fairfax Hospital during the first two years of COVID-19 to the three years prior. We also compared the number of TBI surgeries performed at our institution during the four months of increased lockdown restrictions (March to June 2020) to the same four months of the previous three years. We compared the age of patients receiving surgery for TBI during the first two years of the COVID-19 lockdown to the three years prior. We also compared the age of patients receiving surgery for TBI during the four months of increased lockdown restrictions (March to June 2020) to the same four months of the previous three years. All of these comparisons were performed using t-test statistics.

We further determined if there was a correlation between the number of new COVID-19 cases in Virginia and Northern Virginia with the incidence of performed TBI surgeries four weeks later, or if there was a correlation between the number of new COVID-19 cases in Virginia and Northern Virginia with the average age of patients receiving surgery for TBI four weeks later during the first two years of the COVID-19 pandemic. Our correlation was between the average incidence of COVID-19 and TBI injury data of the following four weeks because we assumed that it would be time for people to be aware of an increase in COVID-19 infections and change their behaviors. For this comparison, we used publicly available data from the State of Virginia on the incidence of new COVID-19 infections in the State of Virginia and the Northern Virginia area organized by four-week periods from April 2020 to February 2021. The data from Inova Fairfax Hospital on the number of TBI surgeries and the average age of TBI surgery patients were also organized by four-week periods from April 2020 to February 2021. We used Pearson correlation statistics for this analysis. Statistics were completed using Minitab Statistical Software (Minitab, LLC, State College, Pennsylvania, United States of America) on a Hewlett-Packard personal computer (HP, Palo Alto, California, United States). Values of p<0.05 were considered statistically significant.

## Results

**Figure 1:**
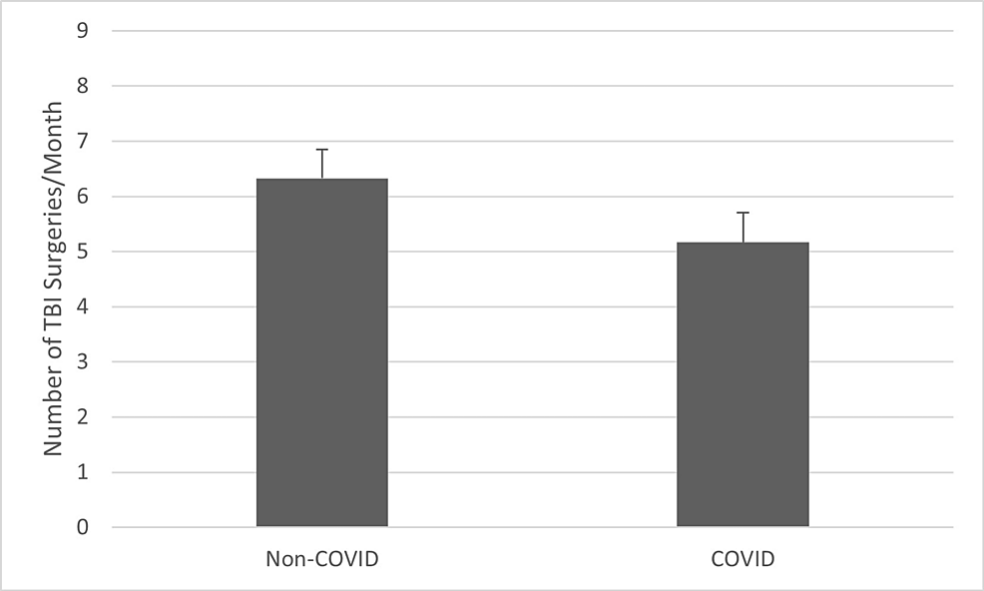
Average number of TBI surgeries performed each month during the three years prior to the COVID-19 pandemic compared to the two years following its start (p>0.05). P-values less than 0.05 were considered statistically significant TBI: traumatic brain injury

**Figure 2:**
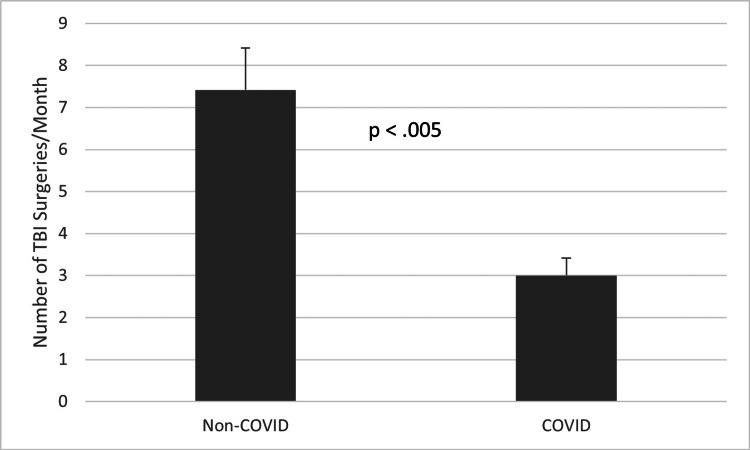
Average number of TBI surgeries performed during the four-month period of increased lockdown restrictions compared to the same four-month period in the prior three years (p<0.005). P-values less than 0.05 were considered statistically significant TBI: traumatic brain injury

A comparison of the average number of performed TBI surgeries per month during the first two years of the COVID-19 pandemic to the average number of performed TBI surgeries per month during every month of the three years prior can be seen in Figure 1. On average, there was approximately one fewer TBI surgery per month during the first two years of the COVID-19 pandemic, but this difference did not reach statistical significance (t=-1.56, p>0.05). However, when comparing the four-month period of increased COVID-19 lockdown restrictions (March 2020 through June 2020) to the same four months (March through June) of the three years prior as seen in Figure 2, there was a much greater decrease in the average number of performed TBI surgeries per month. On average, there were more than four fewer performed TBI surgeries per month during the COVID-19 pandemic, a difference which was statistically significant (t=-4.03, p<0.005). Taken together, these results indicate that the decrease in the average number of TBI surgeries performed per month during the COVID-19 pandemic was likely related to the increased COVID-19 lockdown restrictions.

**Figure 3:**
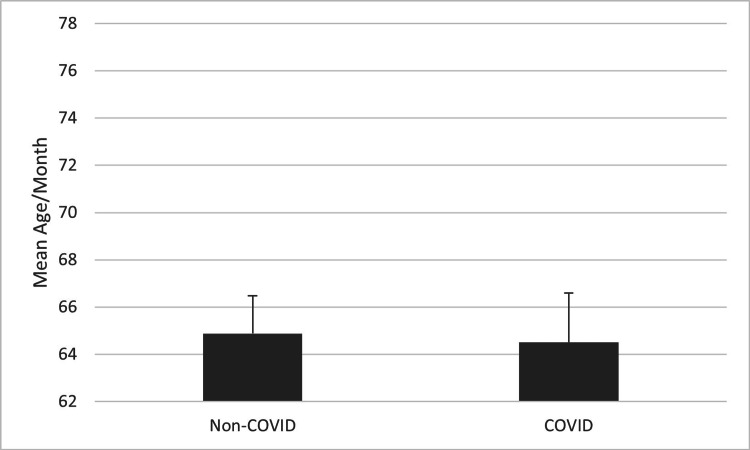
Mean age of patients receiving TBI surgery during the two years after the start of the COVID-19 pandemic compared to the three years prior (p>0.05). P-values less than 0.05 were considered statistically significant

**Figure 4:**
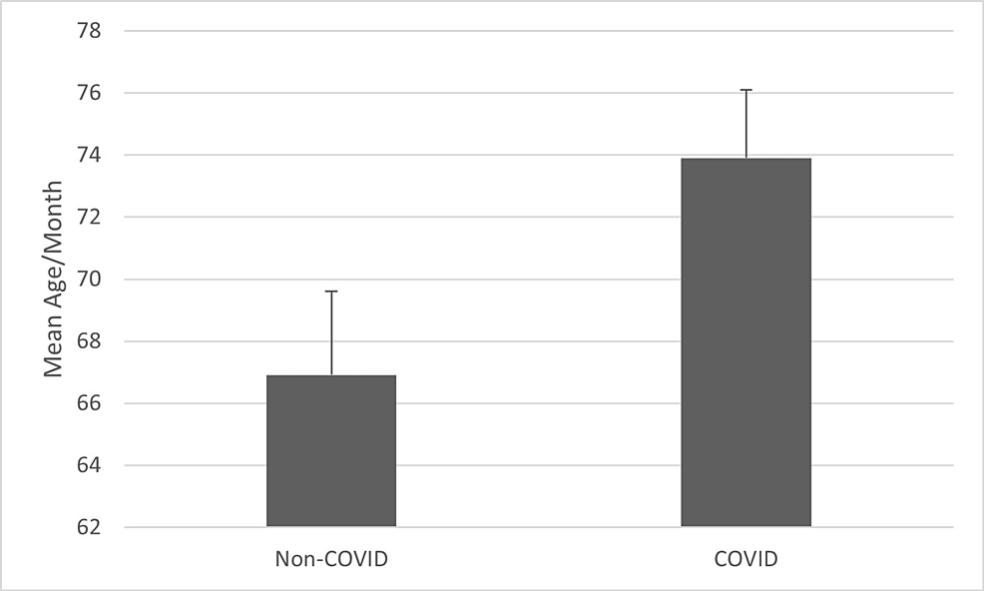
Mean age of patients receiving TBI surgery during the four-month period of increased COVID-19 lockdown restrictions compared to the same four months in the three years prior (p=0.073). P values less than 0.05 were considered statistically significant.

A similar result was seen when assessing the average age of patients receiving TBI surgery, but this comparison did not reach statistical significance. Figure 3 compares the mean age of patients receiving TBI surgery in the first two years following the start of the COVID-19 pandemic to the three years prior. The mean age of the two groups is nearly identical (t=-0.14, p>0.05). A difference becomes apparent when comparing the four-month period of increased COVID-19 lockdown restrictions to the same four months of the three years prior. Figure 4 shows this difference: an over three-year increase in the mean age of patients receiving TBI surgery during the four-month period of increased COVID-19 lockdown restrictions. This was a trend that did not reach statistical significance (t=1.99, p=0.073).

**Figure 5:**
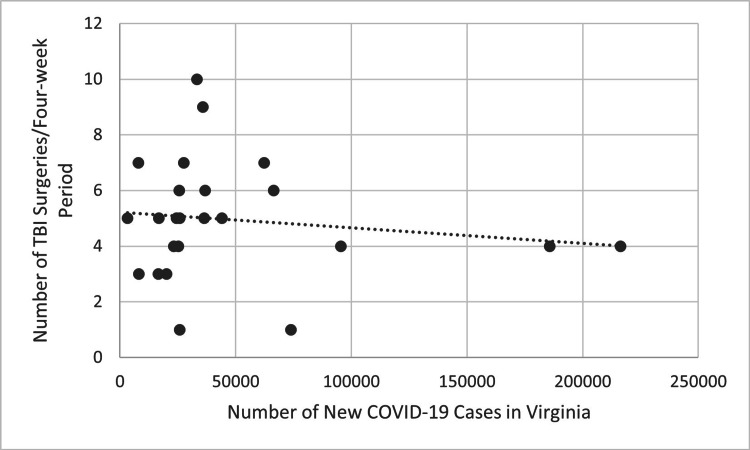
Number of new COVID-19 cases for each four-week period in Virginia compared to the number of TBI surgeries performed four weeks later during the first two years of the COVID-19 pandemic (p>0.05). P-values less than 0.05 were considered statistically significant TBI: traumatic brain injury

**Figure 6:**
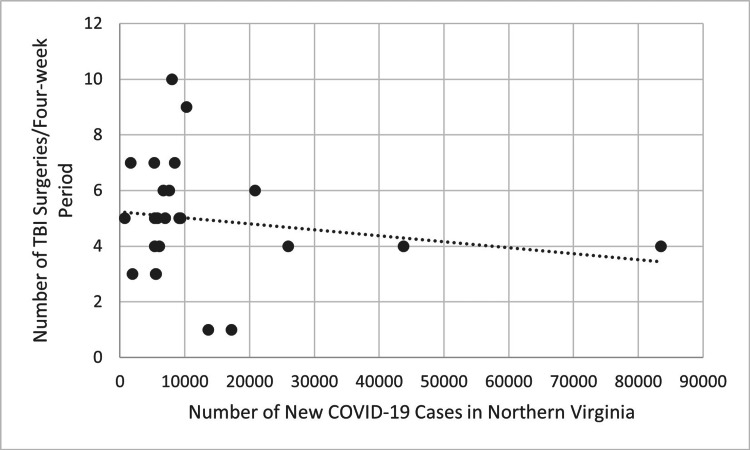
Number of new COVID-19 cases for each four-week period in Northern Virginia compared to the number of TBI surgeries performed four weeks later during the first two years of the COVID-19 pandemic (p>0.05). P-values less than 0.05 were considered statistically significant TBI: traumatic brain injury

Figure 5 compares COVID-19 cases in each four-week block during the first two years of the pandemic to the number of performed TBI surgeries in the following four-week blocks using COVID-19 data for the entire state of Virginia. Figure 6 shows the same comparison using COVID-19 data from only Northern Virginia. The trend line in both figures is decreasing, suggesting a decrease in the number of performed TBI surgeries as the incidence of new COVID-19 infections increased. However, this was not statistically significant using Pearson correlation (R=-0.137, p>0.05 for Virginia, R=-0.177, p>0.05 for Northern Virginia).

**Figure 7:**
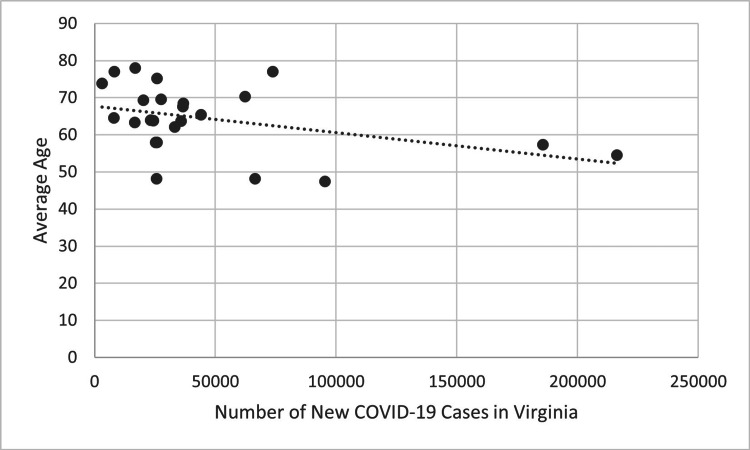
New COVID-19 cases of each four-week period in Virginia compared to the average age of patients receiving TBI surgery four weeks later during the first two years of the COVID-19 pandemic (p<0.05). P-values less than 0.05 were considered statistically significant

**Figure 8:**
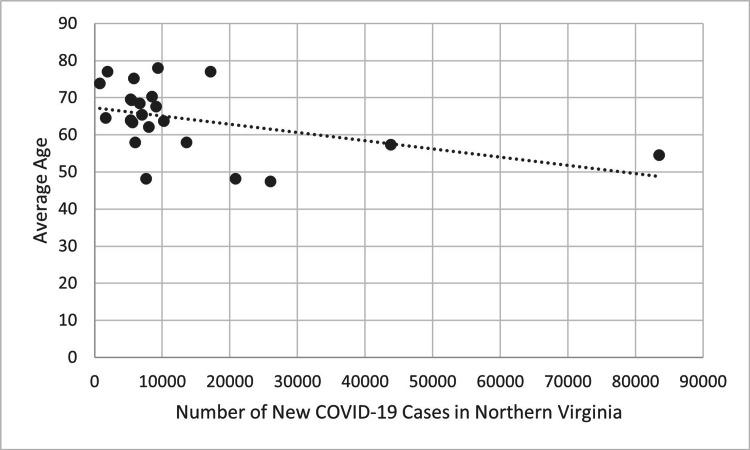
New COVID-19 cases of each four-week period in Northern Virginia compared to the average age of patients receiving TBI surgery four weeks later during the first two years of the COVID-19 pandemic (p<0.05). P-values less than 0.05 were considered statistically significant

There was a statistically significant negative correlation when evaluating the mean age of patients receiving TBI surgery. Figure 7 compares COVID-19 cases in each four-week block during the first two years of the pandemic to the mean age of patients receiving performed TBI surgery in the following four-week blocks using COVID-19 data for the entire state of Virginia. Figure 8 shows the same comparison using COVID-19 data from only Northern Virginia. Both figures show a negative trend line, indicating that the mean age of patients receiving TBI surgery decreased four weeks after the incidence of new COVID-19 cases increased. This trend was statistically significant when using data on the number of new COVID-19 cases in Virginia (R=-0.414, p<0.05) and Northern Virginia (R=-0.433, p<0.05).

## Discussion

Our analysis revealed that during the peak four-month period of COVID-19 lockdown restrictions, there was a marked decline in TBI surgeries compared to the averages of the same four months over the preceding three years. We believe that this decrease in TBI surgeries performed during lockdown restrictions might be a byproduct of fewer opportunities for people to engage in activities that may be associated with TBI such as driving. A study done in Normandy, France, found decreased ICU admissions for TBI during the COVID-19 pandemic, linking it to a 74% decrease in motor vehicle accidents across France, but this study also found an increase in TBI surgery [17]. However, another study found a 49.6% decrease in TBI referrals at a level one trauma center in England, also linking this decrease to a worldwide decrease in road traffic accidents (RTA) due to increased lockdown restrictions [14]. A study done using data from the University Hospital of Wales observed a significant decrease in RTA-associated TBI referrals, a statistic that is much in line with our hypothesis [15]. A 39.5% decrease in concussion visits-another form of TBI-was observed during the first year of the COVID-19 pandemic at a primary clinic in Pittsburgh, Pennsylvania [13]. Contrary to our hypothesis, this study found an increase in concussions from motor vehicle collisions [13]. We believe Pittsburgh’s urban environment and its resulting motor vehicle dependency to be responsible for this discontinuity. Similar to our findings, a study done in the Gyeonggi Province, Korea, found a decrease in the total number of neurosurgery emergency patients during the first nine months of the pandemic [18].

Other studies showed more variance from our findings. A meta-analysis of 13 studies found a higher mortality rate from TBI during the pandemic but only in low- and middle-income countries [24]. The authors attribute this finding to unavailable resources in these countries and an increased incidence of COVID-19 compared to high-income countries, both factors which differ from the Northern Virginia area [24]. A study done in Tyrol, Austria, which showed an increase in head and face injuries, possibly correlating with TBI, also observed a decrease in the number of patients admitted for all types of traumas [25]. Another study done using data from six level-one trauma centers in the United States observed an increase in head injuries during the first year of the pandemic [22]. However, this study also observed a decrease in motor vehicle injuries, which aligns with our hypothesis [22]. A study from Atlanta, Georgia, found an increase in firearm injuries to the head and neck during the COVID-19 pandemic, but this represents a subpopulation of overall TBI which may account for the difference from our findings [21]. A French study found no difference in the incidence of subdural hematoma from child abuse in patients two years old and younger during the pandemic compared to the pre-pandemic period [27]. However, this group is a very specified population of TBI which is likely responsible for this study’s difference in findings compared to ours. A study of traumas treated at four hospitals in Finland found no difference in the incidence of severe injuries or the proportion of head injuries during the COVID-19 pandemic compared to prior years [26]. The authors attributed these findings to more risky behaviors on less crowded roads, which may differ from behaviors in the Northern Virginia area [26].

Our analysis indicated a notable shift toward an older demographic, with the average age of patients undergoing TBI surgery during the heightened COVID-19 lockdown months being 73.9, compared to an average of 66.9 in the same period over the previous three years. We believe this trend to be related to fall-induced TBIs common among elderly patients. While elderly patients continued to be susceptible to TBI-prone falls which frequently occur at home, during the four months of increased lockdown restrictions, patients of younger age groups were less susceptible to TBI-prone activities, such as operating motor vehicles, than they were during the three years prior. A study done in the Netherlands found a similar trend of increased age among TBI patients while lockdown restrictions were in place, linking it to a decrease in commuting among younger populations that might pose the risk of TBI [11]. Furthermore, another study done in Pennsylvania found the mechanism of injury for neurotrauma during the pandemic to be more likely caused by falls than motor vehicle collisions [12].

During the two years following the start of the COVID-19 pandemic, as the incidence of new COVID-19 cases in Virginia and Northern Virginia increased, we found that the average age of patients receiving TBI surgery decreased. We believe that this negative correlation might be a result of increased domestic abuse, especially among children. A study done on the rise of abusive head trauma (AHT) supports this idea, reporting an increase in AHT among children at their institution during the pandemic and linking it to the increased exposure of children to their parents [23].

Another study that agrees with our overall findings observed a decrease in the mean number of TBI cases during the pandemic, linking it to a decrease in traffic accidents [19]. However, this study also observed a decrease in fall-mediated TBI, which is contrary to our hypothesis concerning differences in the ages of people receiving TBI surgery. We believe that this disparity is due to a difference in the periods of time of our respective analyses. While this study drew its conclusion from 400 days during the COVID-19 pandemic, the period in which we observed an increase in age among patients undergoing TBI surgery was only four months. Moreover, these four months were the period of maximum lockdown restrictions.

Another study done in Vento, Italy, observed an overall decrease in the incidence of TBI (approximately 49% less than expected TBI rates) in the year following the start of the COVID-19 pandemic [16]. This is congruent with our findings. Nevertheless, a study in Indonesia observing the same four-month period that we analyzed (March to June 2020) found an increase in hospitalizations from TBI during COVID-19 [20]. This study also observed an increase in road traffic injuries during these four months. The difference in our findings is likely due to the varying compliance with COVID-19 restrictions in each respective region.

A primary limitation of our study is its retrospective design. Retrospective analyses, while valuable, might not account for all potential confounders as effectively as prospective studies. This may affect the generalizability and causative interpretations of our results. Another limitation of this study is that it does not compare the several years prior to the COVID-19 pandemic with each other to see if there was a preexisting, long-term, negative trend in TBI surgeries performed. An overall yearly decrease in operative TBI may inappropriately suggest this trend to be a result of the COVID-19 pandemic as the trend continues through the years analyzed following the emergence of COVID-19. Another limitation of this study is that it uses data from only one hospital in one country while attempting to draw conclusions about a global pandemic. As we have discussed above, other regions of the world have reported results different from our findings suggesting limitations in generalizability. It’s also essential to note that our study centered primarily on operative TBIs. Thus, while our findings shed light on this subset of TBI cases, extrapolating these conclusions to encompass the entire TBI spectrum during the pandemic would require a more expansive analysis.

## Conclusions

With our study, we found an overall decrease in TBI surgeries performed and an increase in the average age of patients receiving TBI surgery during the four months of increased COVID-19 restrictions (March to June 2020). Our findings represent an example of how policy decisions and peoples’ behaviors in reaction to environmental pressures can affect unrelated health conditions such as head trauma. This is important for us to keep in mind when making policies and addressing the concerns of the overall population. This also suggests that changes in society occurring during the COVID-19 lockdown that could be continued after the pandemic, such as an increase in working from home, may have the effect of decreasing severe TBI.
